# Beyond aesthetic outcomes: psychodermatological benefits of botulinum toxin treatment in the upper facial third. A narrative review

**DOI:** 10.3389/fpsyt.2026.1822916

**Published:** 2026-06-09

**Authors:** Álvaro Loewen, Sabina Aranda-Guerrero, Kazuhiro Tajima-Pozo

**Affiliations:** 1 Department of Psychiatry, University Hospital Fundación Jiménez Díaz, Madrid, Spain; 2 Department of Family and Community Medicine, University Hospital Puerta de Hierro Majadahonda, Madrid, Spain; 3 Department of Psychiatry, University Hospital Fundación Alcorcón, Madrid, Spain; 4 Department of AMIR Aesthetic Medicine, Healthcademia Education Group, Madrid, Spain

**Keywords:** botulinum toxin, facial feedback, psychiatric disorders, psychodermatology, skin-brain axis

## Abstract

**Background:**

Botulinum toxin type A (BoNT-A) is widely used in aesthetic dermatology, particularly for dynamic wrinkles in the upper facial third. Beyond cosmetic effects, BoNT-A may influence emotional processing through the skin-brain axis. This narrative review synthesizes evidence on psychological and psychiatric outcomes of upper facial BoNT-A treatment, with a focus on mechanisms extending beyond aesthetics.

**Methods:**

A structured search was conducted in PubMed, Scopus, and Web of Science through January 2026. Search terms included “botulinum toxin,” “upper facial third,” “psychodermatology,” “facial feedback,” “depression,” “anxiety,” and “psychiatric disorders.” Original studies, randomized controlled trials (RCTs), observational studies, systematic reviews, and meta-analyses assessing psychological, psychiatric, or neurobiological outcomes were included.

**Results:**

Upper facial BoNT-A is associated with clinically meaningful reductions in depressive and anxiety symptoms, and improvements in self-perception and social functioning. Inhibition of corrugator and procerus muscles modulates afferent signaling to limbic structures, supporting the facial feedback hypothesis. Neuroimaging evidence demonstrates attenuated amygdala activation after BoNT-A treatment. Psychosocial improvements may reinforce these neurobiological effects. Evidence in bipolar disorder and personality disorders remains limited and methodologically heterogeneous. Caution is advised in patients with body dysmorphic disorder.

**Conclusions:**

BoNT-A in the upper facial third provides psychodermatological benefits beyond aesthetics, improving emotional regulation and affective symptoms. It should be considered a complementary intervention within a biopsychosocial framework. Future large-scale RCTs with standardized psychiatric and neurobiological endpoints are warranted.

## 1 Introduction

Botulinum toxin type A (BoNT-A) has become established as one of the most widely used medical treatments worldwide, especially in aesthetic dermatology (1). In the cosmetic field, its use in the upper facial third, primarily for the treatment of glabellar, forehead, and periorbital lines, represents one of the most frequent minimally invasive interventions in dermatology and aesthetic medicine (2, 3). Beyond its demonstrated efficacy in reducing dynamic wrinkles and improving facial appearance, the impact of botulinum toxin treatment transcends the purely aesthetic realm, influencing emotional, psychological, and social dimensions of the patient (2–4).

In recent decades, the perception of facial aesthetics has acquired growing relevance in quality of life, self-esteem, and social interaction. The face constitutes a central element in emotional communication and in the construction of personal identity, such that alterations in its expression can have significant repercussions on psychological well-being (5). In this context, the improvement of facial appearance through aesthetic treatments is associated not only with visible physical changes, but also with modifications in self-perception, mood, and personal confidence (2–4).

Concurrent with the expansion of aesthetic medicine, there has emerged a growing body of evidence supporting bidirectional communication between the skin and the central nervous system, conceptualized within the skin-brain axis and the field of psychodermatology (6, 7). This interdisciplinary field studies how psychological factors can influence cutaneous diseases and, conversely, how dermatological conditions affect mental health (6–8). Various dermatological disorders have been associated with anxiety, depression, chronic stress, and body image disturbances, reinforcing the notion that the skin acts not only as a physical barrier, but also as a neuroimmunoendocrine organ closely linked to emotional processes (7, 8).

Within this framework, botulinum toxin has attracted particular interest for its potential psychodermatological effects. Previous studies have suggested that treatment with BoNT-A, especially in the upper facial third, may be associated with improvements in depressive symptoms (9–13), anxiety (10, 14, 15), and quality of life (2–4). One of the most explored hypotheses is the facial feedback theory, according to which the modulation of facial muscle activity could influence emotional experience by altering afferent signals to the limbic system (5, 16, 17). Additional mechanisms have been proposed involving the reduction of perceived stress, improvement of self-image, and modification of social interaction patterns (2, 4).

Despite the growing literature on the psychological effects of botulinum toxin, the available findings are dispersed (11–13, 18) and, at times, focused on specific populations or particular psychiatric disorders (10, 14, 19–23). Furthermore, many studies prioritize aesthetic outcomes (3, 4, 20), relegating potential psychodermatological benefits (2) and the underlying mechanisms of the skin-brain axis to a secondary role (6–8, 24).

The objective of this narrative review is therefore to comprehensively analyze the available evidence on the psychodermatological benefits of BoNT-A treatment in the upper facial third, encompassing mechanisms of skin-brain interaction, the facial feedback pathway, and clinical outcomes across major psychiatric domains. This integrative perspective aims to contribute to a broader understanding of BoNT-A within the framework of contemporary psychodermatology.

## 2 Methods

This study was conducted as a narrative review of the literature addressing the psychodermatological effects of BoNT-A applied to the upper facial third. A structured search was performed in PubMed, Scopus, and Web of Science for articles published through January 2026, using combinations of the following terms: “botulinum toxin,” “upper facial third,” “psychodermatology,” “skin-brain axis,” “facial feedback,” “depression,” “anxiety,” and “psychiatric disorders”.

Eligible studies included original research articles, randomized controlled trials (RCTs), observational studies, systematic reviews, and meta-analyses that evaluated psychological, psychiatric, or neurobiological outcomes following BoNT-A injections in the upper facial third. Studies were excluded if they focused exclusively on therapeutic (non-aesthetic) indications unrelated to facial expression, or if they reported no quantifiable psychological outcome.

The literature review was conducted independently by two authors (A.L. and S.A.G.), who performed the search, screening, and data extraction. Findings were cross-checked to ensure accuracy and completeness.

The selected literature was analyzed qualitatively and organized into four thematic domains: mechanisms related to the skin-brain axis and psychodermatology, facial feedback theory and emotional modulation, clinical applications and muscle anatomy, and psychiatric outcomes stratified by diagnostic category. Narrative synthesis was conducted given the heterogeneity of study designs and outcome measures, which precluded formal meta-analytic pooling.

## 3 Relevant sections

### 3.1 The skin-brain axis and psychodermatology

The skin-brain axis constitutes a broad conceptual framework that describes the bidirectional communication between the central nervous system and the skin, mediated by neuroendocrine, immunological, and peripheral pathways (6–8). The skin functions not only as a physical barrier but also as a neuroimmunoendocrine organ capable of perceiving and transmitting emotional signals (8, 24). Specialized cutaneous receptors capture tactile, painful, or temperature stimuli and transmit them via afferent fibers to the spinal cord and brainstem, connecting with limbic system structures such as the amygdala and cingulate cortex, which process emotion and emotional memory (25). Additionally, the skin can release neurotransmitters and cytokines that modulate inflammation, pain perception, and stress response (8, 24). Psychodermatology studies these interactions and how psychological factors can influence cutaneous diseases, as well as how skin alterations affect mental health (6, 7). This framework provides the foundation for understanding how interventions that modulate facial expression, such as botulinum toxin, can impact not only appearance but also the organism’s emotional and stress circuits (24).

### 3.2 Facial feedback and emotional modulation

Within the skin-brain axis, the facial feedback theory offers a specific mechanism by which facial muscle activity contributes to the generation and regulation of emotions (2, 26). The muscles of the forehead and glabellar region, when contracted, send proprioceptive information through the trigeminal nerve to brainstem nuclei and limbic system structures, including the amygdala, insula, and prefrontal cortex, modulating the perception of emotional states such as anger, tension, or sadness (16, 27). The temporary inhibition of these muscles through botulinum toxin alters these afferent stimuli, decreasing the activation of limbic circuits associated with negative emotions and favoring more relaxed and positive emotional states (17). This neurophysiological mechanism demonstrates that the modification of facial expression is not limited to aesthetic effect but directly influences emotional experience and the regulation of anxiety and mood (28–30).

### 3.3 Botulinum toxin in the upper facial third: mechanisms and clinical applications

Botulinum toxin type A acts by blocking the release of acetylcholine at the neuromuscular junction, causing temporary paralysis of innervated muscles (31, 32). In the upper facial third, it primarily affects the corrugator, frontalis, and orbicularis oculi muscles, responsible for dynamic expression lines in the forehead, glabella, and periocular region (33, 34). These structures not only contribute to aesthetics but also participate directly in emotional expression and in the transmission of proprioceptive information to the limbic system, integrating the functional dimension with aesthetics (35). The precise application of the toxin allows modulation of muscle activity without compromising natural expression, optimizing the balance between visual effects and influence on emotional circuits (33). Factors such as dose, injection technique, and patient selection are fundamental to maximizing results and minimizing adverse effects (34, 35).

### 3.4 Psychological and psychiatric outcomes associated with botulinum toxin treatment

Botulinum toxin type A applied to the upper facial third has been associated with psychological effects that extend beyond aesthetic improvement, particularly in psychiatric conditions involving affective dysregulation, anxiety, altered self-perception, and emotional reactivity (2, 11, 18, 36). Although BoNT-A is not indicated as a primary treatment for psychiatric disorders, accumulating evidence suggests that modulation of facial muscle activity may influence emotional processing and social behavior through mechanisms related to the skin-brain axis and facial feedback pathways (11, 13, 36).

Key clinical studies evaluating the psychiatric effects of BoNT-A across diagnostic categories are summarized in **Table 1**, including randomized controlled trials, observational studies, and exploratory designs. Overall, the available evidence suggests potential benefits in depressive and anxiety-related outcomes, as well as in domains of emotional regulation and social functioning. However, the literature is characterized by important methodological limitations, including small sample sizes, short follow-up periods, heterogeneity in study populations and outcome measures, and a predominance of exploratory or non-controlled designs outside depressive disorders.

**Table 1:** Clinical studies of BoNT-A in psychiatric disorders: design, findings, and limitations.

Study	Disorder	Design/N	Main finding	Main limitation
Depression				
Crowley et al., 2022 (9).	Depression	Meta-analysis (9 studies)	BoNT-A improved depression scores versus placebo	Limited number of trials and short follow-up
Shu et al., 2024 (22)	Depression	RCT (N=140)	Higher BoNT-A doses showed greater antidepressant effects	No placebo arm
Ceolato-Martin et al., 2024 (38)	Treatment-resistant depression	RCT (N=58)	Glabellar injections showed stronger antidepressant effects	Small sample size
Anxiety Disorders				
Wollmer et al., 2021 (15)	Anxiety	Pharmacovigilance study	Association with reduced anxiety-related outcomes	Retrospective observational design
Finzi & Rosenthal, 2019 (39)	Social anxiety disorder	Case series (N=6)	Reduction in social anxiety symptoms	Very small uncontrolled study
Borderline Personality Disorder				
Wollmer et al., 2022 (23)	Borderline personality disorder	RCT (N=54)	Reduced emotional instability and depressive symptoms	Small sample size
Schulze et al., 2023 (21)	Borderline personality disorder	fMRI study (N=45)	Altered emotion-related brain connectivity after BoNT-A	Exploratory design
Kruger et al., 2022 (36)	Borderline personality disorder	Experimental study (N=45)	Altered emotional processing after treatment	Small sample size
Other Exploratory Indications				
Finzi et al., 2018 (19)	Bipolar depression	Case series (N=6)	Improvement in depressive symptoms	Very small uncontrolled study

BoNT-A, Botulinum toxin type A; RCT, randomized controlled trial; fMRI, functional magnetic resonance imaging.

These limitations warrant cautious interpretation and highlight the need for larger, well-designed randomized controlled trials with standardized psychiatric endpoints.

Given the variability in the strength and quality of evidence across diagnostic categories, the following sections summarize findings accordingly.

#### 3.4.1 Depressive disorders

Evidence from meta-analyses, systematic reviews, randomized controlled trials, and observational studies has reported reductions in depressive symptom severity following BoNT-A injections in the glabellar region (9–11, 13, 18, 22, 37).

The proposed mechanism centers on the facial feedback hypothesis, whereby inhibition of corrugator and procerus muscle activity reduces negative afferent signaling to limbic structures such as the amygdala and insula (2, 36). Functional neuroimaging studies have demonstrated attenuated amygdala activation during the processing of negative emotional stimuli after BoNT-A treatment, supporting a neurobiological basis for mood modulation (36).

Evidence indicates that the specific site of injection may influence emotional outcomes, with differences observed between glabellar, frontalis, and combined applications. This highlights the importance of anatomical targeting within the facial feedback pathway (38).

Additional contributory factors include improvements in self-image, reduced perception of negative social feedback, and enhanced interpersonal engagement (2, 11). Despite these findings, heterogeneity in study design and sample characteristics necessitates cautious interpretation, and BoNT-A should be regarded as a complementary intervention rather than a standalone antidepressant therapy (13, 18).

#### 3.4.2 Anxiety disorders

Several studies have reported reductions in anxiety-related symptoms following botulinum toxin treatment in the upper facial third (10, 15, 18).

Decreased activation of facial muscles associated with fear, concern, or anger may reduce afferent input to limbic and autonomic centers involved in anxiety regulation, contributing to subjective feelings of calm and emotional control (5, 16, 17).

Regarding Social Anxiety Disorder, by softening expressions associated with tension or worry, BoNT-A treatment may reduce perceived social threat and facilitate more positive interpersonal interactions (3, 14). Improved facial appearance and expressivity may also influence social feedback loops, reinforcing self-confidence and reducing avoidance behaviors (3, 5). Beyond these mechanisms, preliminary observations suggest that BoNT−A may directly alleviate social anxiety symptoms in broader populations. Improvements in interpersonal functioning and perceived social threat have been reported, although data remain limited and require confirmation in controlled studies (39). While direct evidence remains limited, these mechanisms suggest a plausible adjunctive role for botulinum toxin in socially mediated anxiety symptoms (15, 18).

#### 3.4.3 Bipolar disorders

Potential benefits of BoNT-A in bipolar disorder may relate to its effects during depressive phases, particularly through facial feedback modulation and improvements in self-perception (11, 12, 19).

Evidence on botulinum toxin in manic or hypomanic states is limited, and safety data for patients with bipolar disorder remain scarce; therefore, its use should be cautious and restricted to settings with psychiatric monitoring and mood stabilization (11, 12, 19).

#### 3.4.4 Personality traits and disorders

Theoretical models suggest that modulation of facial feedback may influence emotional intensity and interpersonal signaling in individuals with certain personality traits (2, 11, 16). By reducing exaggerated facial expressions associated with anger, distress, or tension, BoNT-A may contribute to improved emotional regulation and social interaction in selected cases (18, 23, 36).

However, patients with personality traits may present increased vulnerability to unrealistic expectations or dissatisfaction with aesthetic outcomes (37, 40). Consequently, the use of botulinum toxin in this population requires heightened clinical caution, clear therapeutic boundaries, and close collaboration with mental health professionals (18, 23).

#### 3.4.5 Body dysmorphic disorder

Particular caution is warranted in patients with body dysmorphic disorder (BDD), as these individuals frequently seek cosmetic procedures, including botulinum toxin type A, yet these interventions rarely result in sustained psychological improvement (41, 42). Moreover, the presence of unrealistic expectations or excessive preoccupation with perceived defects has been associated with persistent dissatisfaction, repeated treatment-seeking, and poor psychosocial outcomes in aesthetic populations (20, 37, 40).

Therefore, BoNT-A should not be considered a therapeutic strategy for BDD, and careful psychiatric evaluation and referral are essential when body image pathology is suspected (3, 41).

## 4 Discussion

This narrative review highlights that botulinum toxin type A (BoNT−A) injections in the upper facial third exert psychodermatological effects that extend beyond aesthetic enhancement, influencing emotional processing, affective symptoms, and social functioning.

Evidence from randomized controlled trials (**Table 1**) suggests that BoNT-A may reduce depressive symptom severity, with several studies demonstrating statistically significant improvements compared with placebo (9–11, 13). Meta−analyses further support a modest but consistent antidepressant effect (11, 13, 18). However, these findings require cautious interpretation, as most trials are limited by small sample sizes, short follow−up periods, and heterogeneity in inclusion criteria and psychiatric outcome measures (18, 22).

Beyond depression, emerging evidence suggests potential benefits for anxiety−related symptoms, particularly in socially mediated contexts. Observational studies and small controlled trials have reported reductions in anxiety levels and improvements in social confidence following BoNT−A treatment (10, 14, 15), although the strength of evidence remains weaker than that for depressive disorders (18). Data regarding bipolar disorder and personality disorders are preliminary and heterogeneous, consisting mainly of small trials, case series, or exploratory neuroimaging studies (11, 12, 19, 23, 36), which precludes firm conclusions.

Mechanistically, the psychotropic effects of BoNT−A are supported by converging preclinical and human evidence. Preclinical studies demonstrate that modulation of peripheral sensory input can influence central stress and emotional circuits through neuroimmune and neuroendocrine pathways within the skin-brain axis (8, 24, 25). In humans, functional neuroimaging studies provide more direct support: inhibition of corrugator muscle activity via BoNT−A has been associated with reduced amygdala activation during the processing of negative emotional stimuli (16, 17, 36). These findings offer neurobiological validation of the facial feedback hypothesis, suggesting that peripheral modulation of facial musculature can translate into measurable changes in limbic system activity.

Psychosocial mechanisms likely act in parallel with these biological pathways. Improvements in self−perception, attenuation of negative facial expressions, and changes in social feedback loops may reinforce emotional regulation and contribute to the overall therapeutic effect (2, 4, 5). The interplay between neurobiological and psychosocial factors supports a biopsychosocial model of BoNT−A action within psychodermatology.

Despite these promising findings, several methodological limitations must be emphasized. Most randomized controlled trials include small samples, limiting statistical power and external validity (18, 22). Considerable heterogeneity exists across studies in terms of patient populations, injection protocols, and psychiatric outcome measures, complicating direct comparisons (11, 18). Observational studies, while informative, are particularly susceptible to selection bias and confounding (10, 15).

A specific methodological challenge in BoNT−A research is functional unblinding. Because the muscular effects of BoNT−A are visible and perceptible, participants may correctly infer their treatment allocation, introducing expectancy bias and potentially inflating subjective improvements in mood or anxiety (11, 13). This limitation is inherent to the mechanism of action of BoNT−A and must be considered when interpreting placebo−controlled trials.

Clinically, current evidence suggests that BoNT−A may serve as an adjunctive intervention in selected patients, particularly those with mild−to−moderate depressive symptoms, social anxiety features, or heightened emotional reactivity (18). However, it should not replace established psychiatric treatments. Careful patient selection remains essential, especially in individuals with body dysmorphic disorder, maladaptive personality traits, or unrealistic expectations, where aesthetic interventions may be contraindicated or require psychiatric co−management (37, 41).

Finally, integrating psychodermatological competencies into aesthetic medicine practice represents an important opportunity. Clinicians trained to identify psychiatric comorbidities and manage psychosocial expectations may enhance both safety and outcomes in patients undergoing BoNT−A treatment.

Overall, while current evidence supports the psychodermatological relevance of BoNT−A, larger, well−designed randomized controlled trials with standardized psychiatric and neurobiological endpoints are needed to clarify its therapeutic potential and define its role within clinical practice.

## 5 Limitations

Several limitations of the available evidence should be noted. First, the narrative review methodology, while appropriate given the heterogeneity of included studies, does not permit quantitative synthesis of effect sizes or formal assessment of publication bias. Second, included studies vary substantially in design, sample size, patient population, injection site, dosing protocols, and psychiatric outcome measures, limiting direct comparability. Third, most RCTs are small, and many observational studies rely on self-reported outcomes without blinded assessment. Fourth, the absence of standardized psychiatric endpoints across studies makes it difficult to draw definitive conclusions regarding the magnitude and durability of psychodermatological effects. Fifth, the present review was limited to English-language publications, which may introduce language bias. These limitations reinforce the need for large, well-designed, prospective RCTs with standardized psychiatric endpoints as a priority for future research.

## 6 Conclusions

Botulinum toxin type A applied to the upper facial third demonstrates psychodermatological effects that extend meaningfully beyond aesthetic improvement. Current evidence supports its role in modulating emotional processing through facial feedback mechanisms within the skin-brain axis, contributing to reductions in depressive symptoms, attenuation of anxiety, and improvements in self-perception and social functioning. BoNT-A should be considered a complementary intervention within a broader biopsychosocial framework rather than a primary psychiatric treatment. Future large-scale RCTs with standardized psychiatric and neurobiological outcome measures, longitudinal neuroimaging studies, and investigation of dose-response relationships and individual moderators will be essential to consolidate the evidence base and enable more personalized clinical application within psychodermatology.

## 7 Future directions

Future research should prioritize large, well-designed RCTs with standardized protocols and validated psychiatric primary endpoints to clarify the magnitude and duration of psychodermatological effects. Longitudinal neuroimaging studies are needed to further elucidate causal mechanisms linking peripheral muscle modulation to limbic activity over time. Investigation of dose-response relationships, optimal injection intervals, and individual moderators such as baseline symptom severity, personality traits, and biological sex may facilitate more personalized and evidence-based application within psychodermatology.
